# Clinical Profile Associated with Adverse Childhood Experiences: The Advent of Nervous System Dysregulation

**DOI:** 10.3390/children4110098

**Published:** 2017-11-15

**Authors:** Jorina Elbers, Cynthia R. Rovnaghi, Brenda Golianu, Kanwaljeet J. S. Anand

**Affiliations:** 1 Department of Neurology and Neurological Sciences, Stanford University School of Medicine, 291 Campus Drive, Stanford, CA 94305, USA; jelbers@stanford.edu; 2 Pain/Stress Neurobiology Laboratory, Department of Pediatrics, Stanford University School of Medicine, 291 Campus Drive, Stanford, CA 94305, USA; crovnagh@stanford.edu (C.R.R.); anandam@stanford.edu (K.J.S.A.); 3 Department of Anesthesiology, Perioperative & Pain Medicine, Stanford University School of Medicine, 291 Campus Drive, Stanford, CA 94305, USA; bgolianu@stanford.edu; 4 Departments of Pediatrics, Anesthesiology, Perioperative & Pain Medicine, Stanford University School of Medicine, 291 Campus Drive, Stanford, CA 94305, USA

**Keywords:** child, nervous system, psychosomatic, stress, adverse childhood experiences

## Abstract

Background: We report the prevalence of children with multiple medical symptoms in a pediatric neurology clinic, describe their symptom profiles, and explore their association with adverse childhood experiences (ACEs). Methods: We retrospectively reviewed 100 consecutive patients from an outpatient pediatric neurology clinic. Patients were included if they were ≥5 years old and reported ≥4 symptoms that were unexplained for ≥3-months. Symptom profiles across six functional domains were recorded: (1) executive dysfunction, (2) sleep disturbances, (3) autonomic dysregulation, (4) somatization, (5) digestive symptoms, and (6) emotional dysregulation. ACEs were scored for all patients. Results: Seventeen patients reported ≥4 medical symptoms. Somatization, sleep disturbances, and emotional dysregulation occurred in 100% patients, with executive dysfunction (94%), autonomic dysregulation (76%), and digestive problems (71%) in the majority. Forty-two children reported ≥1 ACE, but children with ≥4 symptoms were more likely to report ACEs compared to other children (88% vs. 33%; *p* < 0.0001) and had a higher median total ACE score (3 vs. 1; *p* < 0.001). Conclusions: Children with multiple medical symptoms should be screened for potential exposure to ACEs. A clinical profile of symptoms across multiple functional domains suggests putative neurobiological mechanisms involving stress and nervous system dysregulation that require further study.

## 1. Introduction

Pediatricians frequently see patients with symptoms for which no organic disorder can be found, including chronic pain, dizziness, and constipation. Patients with these complaints often have co-morbid sleep problems, cognitive difficulties involving attention and memory, and psychiatric symptoms including depression, anxiety, and panic attacks. Patients may frequently see primary care physicians and multiple medical subspecialists, undergoing numerous and sometimes invasive investigations, which can be frightening for a child, frustrating for the family, and costly to the medical system. 

There is an emerging understanding of intricate links between stress and the development of stress-related medical symptoms. Somatic and autonomic symptoms are commonly reported by combat veterans [1], refugees [2] and abuse survivors [3]. Adults with a history of conversion disorder [4], somatization [5], and migraine [6] have reported high levels of childhood trauma and abuse. Exposure to early life stress can alter neuroendocrine structure and function through neuroplasticity, and may contribute to physical and mental health conditions [7,8]. Early life stressors have been associated with cortisol [9] and autonomic dysregulation [10], in addition to an increased risk of adverse health outcomes ranging from cardiovascular disease and lung disease to cancer [8]. The study of toxic stress is an emerging field of scientific focus, with a recent American Academy of Pediatrics statement calling for science-based efforts in our understanding of childhood adversity [11]. Over a decade of research now suggests that toxic stress causes chronic, dysregulated activation of the stress response system [12,13,14].

Stress activates a cascade of neural and neuroendocrine responses mediated by the autonomic nervous system (ANS) and hypothalamic-pituitary-adrenal (HPA)-axis. Previous studies have identified dysregulation of autonomic and cortisol responses in adults and children with a history of trauma [14,15,16]. Similarly, autonomic and HPA axis dysregulation has been implicated in the pathophysiology of stress-related symptoms such as migraine [17,18], dizziness [19], digestive problems [20], and panic disorder [16]. Medical syndromes of migraine, depression, and post-traumatic stress disorder (PTSD) exhibit a similar list of co-morbid symptoms, suggesting a shared pathophysiology. Despite the critical nature of the ANS and HPA-axis, pediatricians frequently do not assess their function as they fall outside standard laboratory, neurophysiological, and neuroimaging investigations. If ANS and HPA-axis dysregulation is implicated as a cause of medical symptoms, their widespread actions throughout the nervous system would naturally result in symptoms across many of the nervous system’s functional domains.

This observational study aimed to report the prevalence of multiple medically unexplained symptoms within a general pediatric neurology clinic and their association with adverse childhood experiences. We hypothesized that children with 4 or more symptoms would have a higher prevalence of adverse childhood experiences (ACEs) compared to children with less than 4 symptoms or known organic conditions. We also aimed to describe the symptom profile of these children across 6 functional domains within the nervous system: executive function, sleep, autonomic function, somatic function, digestion, and emotional regulation.

## 2. Methods

Following approval by the Institutional Review Board, a retrospective chart review was conducted of 100 consecutive patients over 5 years of age from a weekly pediatric neurology clinic between June–August 2015. A board-certified pediatric neurologist assessed all patients. Children aged 5 to 18 years of age, with at least four medical symptoms lasting longer than three-months, were included. We selected a cut-off of four symptoms in order to identify patients with involvement of multiple functional domains, as opposed to identifying patients with involvement of fewer functional domains who may be more likely to have an organic cause (e.g., familial dysautonomia). Children with moderate-to-severe intellectual disability or developmental delay were excluded, as subjective symptoms are difficult to discern without reliable verbal report. Laboratory testing (complete blood count, metabolic panel, iron studies, thyroid function, erythrocyte sedimentation rate, and anti-nuclear antibody), magnetic resonance imaging of the brain and spine, electroencephalogram, electrocardiogram, and autonomic testing (tilt table) were performed as clinically indicated. Patients with underlying, organic medical conditions who could account for their symptoms were excluded. Electronic patient medical records were reviewed to collect demographics, clinical presentation, prior emergency department or urgent care visits, subspecialty consultations, and investigations related to medical symptoms.

Clinical symptoms per parent and child report were divided into six functional domains of the nervous system: (1) executive dysfunction, (2) sleep disturbances, (3) autonomic dysregulation, (4) somatic symptoms, (5) digestive symptoms, and (6) emotional dysregulation. Executive dysfunction included poor memory, slow processing, or attention problems resulting in impaired school performance. Sleep disturbances included insomnia, frequent night wakings (twice or more per night), or subjective report of frequent nightmares. Autonomic dysregulation included migraine headaches (defined as episodic unilateral or holocephalic moderate-to-severe headache, with associated autonomic symptoms such as nausea, vomiting, photophobia, or phonophobia), dizziness (defined as light-headedness or vertigo), and syncope. Somatic symptoms included chronic pain (tension-type headache or chronic daily headache, abdominal pain, or other body pain) and functional neurological deficits (tremor, weakness, paresthesias, or gait unsteadiness). Digestive problems involved dysfunction of the enteric nervous system including gastro-esophageal reflux, nausea, vomiting, gastroparesis, diarrhea, or constipation. Emotional dysregulation included depression, anxiety, emotional lability, or panic attacks.

The pediatric neurologist (JE) collected a detailed social history in all 100 patients. There is currently no validated tool for collecting ACEs in children; however, the Center for Youth Wellness is currently developing such a tool for health professionals. In accordance with the original ACE study [8], social histories included household members, exposure to domestic violence, substance abuse in the home, parental and peer relationships, family history of psychiatric illness, and history of trauma or abuse. Information obtained from subsequent visits was included in data collection. Children 12 years of age or older were interviewed in private. Disclosure of events that jeopardized a child’s safety were reported to Child Protective Services. ACEs were recorded under nine categories, as reported in prior studies [8,21]: (1) physical abuse, (2) emotional abuse, (3) sexual abuse, (4) emotional or physical neglect, (5) incarcerated household member, (6) exposure to domestic violence, (7) substance user in the home, (8) mental illness in a household member, and (9) being raised by one parent or by adults other than parents. Additional experiences reported as stressful by patients or families were also recorded and considered as an adverse childhood experience; however, these experiences were not included in the total ACE score. To represent cumulative exposure to ACEs within these 9 categories, a total ACE score was calculated for each patient.

### Statistical Analysis

Relationships between the exposure to at least one ACE and each categorical domain of ACEs were compared using Fisher’s Exact test between patients with 4 or more medically unexplained symptoms (group A) and less than 4 medically unexplained symptoms (group B). Univariate analysis was used to report the median total ACE scores of these two groups and compared using two-tailed Mann-Whitney or t-tests. Spearman rank correlation coefficients using a two-tailed test with a confidence interval set at 98% were calculated to determine the associations between the number of symptomatic functional domains, other medical problems, and the total ACE score between the two groups. Association between the number of symptomatic functional domains and the total ACE score across all patients was tested using linear regression. A comparison of fits for regression lines was performed to confirm that different curves must be plotted for the number of symptomatic functional domains and the total ACE score. Statistical analyses were performed using the Prism 7 program (La Jolla, CA, USA) and SAS version 9.3.

## 3. Results

Out of 100 consecutive child neurology outpatients over 5 years of age, 26 children presented with 4 or more symptoms; 6 were excluded because of developmental delay, and 3 patients were excluded because of known organic medical diagnoses (congenital heart disease, moyamoya disease, and autism). A total of 17 patients remained (group A), of whom 12 were females (70%), with a median age of 14 years (range 5–17 years). Of the remaining 83 patients, there were 35 females (42%, *p* = 0.037) with a median age of 11.8 years (range 5–17). All patients in group A exhibited symptoms affecting four or more functional domains, and 53% had involvement in all six domains: executive dysfunction (in 16/17; 94%), sleep disturbances (100%), autonomic dysregulation (in 13/17; 76%), somatization (100%), digestive problems (in 12/17; 71%), and emotional dysregulation (100%). (Table 1). Sleep disturbances, reported in all patients, included insomnia (88%), frequent night wakings (41%), and nightmares (24%). Chronic fatigue was reported only in group A patients (65%; *p* < 0.0001). Other co-morbid conditions included eating disorders (3/17; 18%), tics (3/17; 18%), idiopathic cardiac rhythm abnormalities (3/17; 18%), suicidality (3/17; 18%), postural orthostatic tachycardia syndrome (2/17; 12%), psychogenic non-epileptic events (2/17; 12%), and essential tremor (1/17; 6%).

In group A, 11/17 patients were involved in at least one other subspecialty service (65%), and nine patients were involved in at least three subspecialty services (range 3–5) (53%), including Psychiatry, Pain, Otolaryngology, Gastroenterology, Cardiology, Ophthalmology, Gynecology, and Pulmonology. Eleven patients had at least one visit to the Emergency Department or Urgent Care for their symptoms in the last year (range 1–5 visits) (65%). Ten children had missed at least 10 days of school in the last year due to their symptoms (59%), and 5 patients were on home-hospital or independent study (29%). Ten patients reported that their symptoms regularly interfered with their ability to attend school or participate in extra-curricular activities (59%).

On detailed social history, 42/100 patients reported at least one adverse childhood experience, with 15/17 patients in group A (88%) (Table 1) and 27/83 remaining patients in group B (33%) (*p* < 0.0001) (Table 2). Three patients in group B reported stressful adverse events that were not included in the total ACE score (Table 2). Seven children had an ACE score of 4 or more, and all were in group A (41%). Out of all children who reported one or more ACEs, children with ≥4 medically unexplained symptoms (group A) had a higher median total ACE score of 3 (range 0–6) compared to patients with 0–3 medically unexplained symptoms (group B) (median ACE score = 1, range 0–2) (*p* < 0.0001). (Table 3).

**Figure 1:**
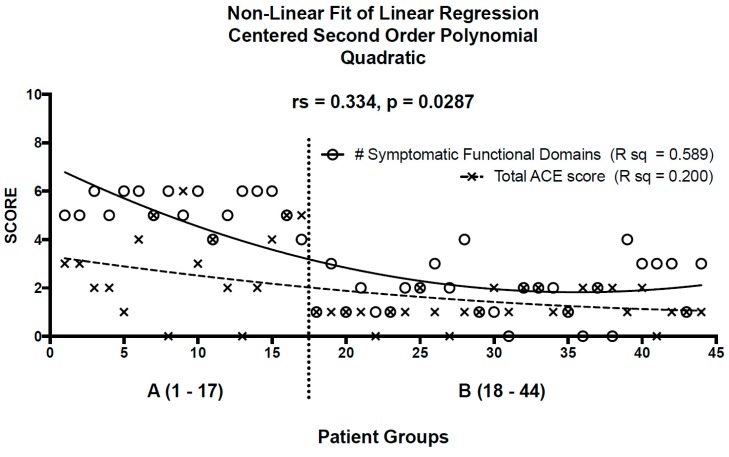
Non-linear fit of linear regression using a centered, second-order polynomial quadratic equation gives rise to lines of best fit for patients in Group A (≥4 medically unexplained symptoms; *n* = 17) and Group B (<4 medically unexplained symptoms; *n* = 27) for the number of symptomatic functional domains (r_s_ = 0.589) and total ACE score (r_s_ = 0.20). There is a moderate and statistically significant correlation between the number of symptomatic functional domains and total ACE score for all patients (r_s_ = 0.334, *p* = 0.0287).

The correlation matrix for patients in both groups revealed positive associations between them: (1) the number of symptomatic functional domains and the prevalence of other medical problems (r_s_ = 0.5, *p* = 0.0002); (2) the number of symptomatic functional domains and the total ACE score (r_s_ = 0.4, *p* = 0.018); (3) the number of other medical problems and the total ACE score (r_s_ = 0.4, *p* = 0.007); and (4) the number of other medical problems and the duration of medical symptoms (r_s_ =0.3, *p* = 0.027). Linear regression using a polynomial quadratic equation gave rise to two lines of best, non-linear fit for patients in group A and group B for: (1) the number of symptomatic functional domains (R sq = 0.589); and (2) the total ACE score (R sq = 0.200). Spearman correlation (r_s_ = 0.354, *p* = 0.018) demonstrated a significant monotonic relationship between the number of symptomatic functional domains and the total ACE score. (Figure 1) The correlation coefficient was strongly influenced by two individuals from Group A, each of whom had 6 symptomatic functional domains with a total ACE score of zero (patients 8 and 13). According to the Rout test for outliers, these were not statistical outliers. Removing these two patients from Group A improved the correlation between number of symptomatic functional domains and total ACE score (r_s_ = 0.525, *p* = 0.0004). These two patients, both females aged 13 and 15 years, may represent underreporting of ACEs, genetic variabilities, or other unidentified factors leading to increased symptomatic functional domains.

## 4. Discussion

Medically unexplained symptoms account for 10–30% of pediatric primary care visits [22] and up to 60% of adult neurology referrals [23]. Comorbidities are common, with many patients exhibiting multiple somatic symptoms, emotional dysregulation, sleep problems, and cognitive complaints, but without a clear explanation or a shared pathophysiology [24]. In our study of 100 consecutive pediatric neurology patients over 5 years of age, 17% patients presented with at least four medically unexplained symptoms for longer than three months. Among these, 29% were being home-schooled, 59% had missed school 10 days or more, and 59% experienced impact on regular school attendance or participation in extracurricular activities. In addition, 65% patients had been seen by at least one other medical subspecialist, and 65% had at least one visit to the Emergency Department within the last year. Patients with multiple medically unexplained symptoms represent a common and fiscally important patient population within our medical and education systems, yet their underlying pathophysiology and most effective treatments are not well understood.

Felitti et al. [8] first demonstrated a link between adverse childhood experiences and adult health risk behaviors and diseases, such as alcoholism, substance abuse, heart disease, and cancer. They further revealed the cumulative impact of ACEs, whereby 4 or more ACEs increased the risk of developing adverse health outcomes. In adults, there can be a lag time of many years, even decades, before early adverse experiences are observed to be associated with adult chronic disease states [14]. While this association is not fully understood, and may reflect currently unidentified confounding factors, the significant correlation between ACEs and health outcomes deserves further investigation into potential pathophysiological mechanisms. 

In children, exposure to early trauma and toxic stress has been associated with learning difficulties, insomnia, eating disorders, asthma, and viral infections [15]. In our study, patients with 4 or more medically unexplained symptoms were similarly more likely to have eating disorders and asthma. Further, patients with this symptom profile were significantly more likely to have a history of adverse childhood experiences and a higher total ACE score. The high prevalence of ACEs in this study suggests that physicians should screen for adverse experiences in children with a clinical profile of multiple medically unexplained symptoms across at least 4 functional domains. The unexplainable nature of these symptoms, and the rising cost of indeterminate investigations and ineffective treatments, suggest that an alternate approach to these patients would benefit both the patient and the health care system. The long-standing and repeated associations between stress and medically unexplained, or psychosomatic, symptoms across decades of research, implicate the stress response system in the generation of these symptoms.

The developing nervous system is particularly susceptible to the effects of extreme or chronic stress. Neuroplasticity adapts to repeated stress responses and establishes a new physiological baseline characterized by structural changes in the brain and dysregulated neural and hormonal responses within ANS, HPA-axis, and sleep/arousal systems [12,25]. Chronic stress leads to elevated levels of corticotropin-releasing hormone, hippocampal cell loss, reduced prefrontal cortical volume, and associated cognitive dysfunction [26]. Children who experience adverse events also develop dysregulated neural responses resulting from chronic or toxic stress. Autonomic testing in adults and children with PTSD demonstrates dysregulated autonomic control, with increased sympathetic and decreased parasympathetic activity [16,27]. Dysregulation of the HPA-axis and ANS is similarly reported in patients with migraine [17,28], dizziness [19], digestive problems [20], sleep disturbance [29], attention deficit hyperactivity disorder [30], and panic disorders [16]. The vagus nerve, traditionally seen as the brake for the sympathetic nervous system, becomes less active during the stress response. This creates an internal system which is unable to effectively maintain its important functions for repair, restoration, and health. When heart rate variability was assessed in the Framingham Heart Study, autonomic dysregulation and decreased vagal function was associated with hyperglycemia [31], risk of new cardiac events [32], and all-cause mortality [33]. Therapeutically, vagal nerve stimulation is a new effective treatment for patients with migraine [34], depression [35] and chronic pain syndromes [36], implicating vagal nerve dysfunction in their pathophysiology. A dysregulated nervous system that has adapted to chronic stress over time with low vagal activity may impair the body’s ability to effectively regulate all the functions of the nervous system including sleep, digestion, autonomic function, motor function, and sensory perception. In the absence of appropriate investigations, the clinical consequences of nervous system dysregulation are likely to present as multiple, medically-unexplained symptoms across multiple subspecialty domains.

This study proposes a novel neurobiological framework for children with multiple medical symptoms as a consequence of ACEs and nervous system dysregulation, but it has important limitations. Retrospective data collection carries with it the inherent risk of missing data, and may lead to the underestimation of reported results. Scientific research in the field of stress is challenging because of the multitude of potential confounders including genetic, environmental, social, and constitutional factors that affect the impact of stress and resilience in each individual. While this study addresses some of the social antecedents that may contribute to medically unexplained symptoms, we were unable to control for genetic and constitutional factors. Arguably, many people experience adversity, trauma, or extreme stress during their lifetime, yet do not develop medical symptoms. Conversely, consequences of a child’s medical problems, poor school performance, or inability to manage emotions may be stressful enough to cause medical symptoms, particularly in the setting of disrupted attachment with a caregiver. It is important to note that nervous system dysregulation describes what is happening at the level of the nervous system in response to stress, without needing to identify “why”, whether it be a particular stressor or an individual vulnerability to stress.

Our research is further limited by the unavailability of a standardized, validated screening tool for assessing a child’s exposure to ACEs. Adults are more likely to report their ACEs history because they have more emotional coping strategies and fewer negative repercussions. While a standardized method of collecting a history of adverse events in children has obvious benefits, it is likely to underestimate traumatic experiences such as emotional, physical, or sexual abuse. Furthermore, standardized tools based on the adult ACEs study may not adequately assess the impact of other negative life experiences such as accidents, hospitalizations, and performance pressure, which can similarly activate a chronic stress response in some children. Often, a trusting therapeutic relationship is the most effective tool in screening for ACEs. A final limitation of this study is the inability to generate a causal link between stress and nervous system dysregulation without directly testing the ANS and HPA-axis. These systems are generally not tested in conventional medical practice. In addition, currently used investigations such as salivary cortisol and heart rate variability may not demonstrate abnormalities under resting conditions. The proposed framework suggests that testing of the ANS and HPA-axis under stress conditions may be warranted in these patients, and requires further study.

## 5. Future Directions

This preliminary study introducing the concept of nervous system dysregulation opens doors to many different research directions for clinicians and scientists. Firstly, it challenges the intellectual paradigm that psychosomatic symptoms are psychological or “in a patient’s head”, and suggests they are instead the result of physiological alterations of an untested biological system. Future studies to test the autonomic nervous system using heart rate variability during resting and challenge conditions, in addition to cortisol responses, are necessary to validate this hypothesis. Studies identifying genetic factors associated with stress or resilience such as FKBP5 polymorphisms [37,38] may help to explain why some children respond to stress in this manner while others remain asymptomatic. Early evidence implicates HPA-axis and autonomic dysregulation in insulin resistance [39], impaired innate immunity [40] and pro-inflammatory responses [41]. Future studies aimed at understanding the neural, hormonal, and immune consequences of stress in the context of nervous system dysregulation may identify novel therapeutic approaches to disease and health promotion.

## 6. Conclusions

Children with ≥4 unexplained medical symptoms are common, accounting for 17% of patients presenting to an outpatient child neurology clinic, and are associated with a higher prevalence of adverse childhood experiences. These symptoms are likely to be related to a nervous system that has adapted to chronic stress, whereby physiological dysregulation impairs the ability to control and effectively regulate all the functions of the nervous system: emotional processing, sleep-wake cycles, autonomic function, digestion, motor function, and sensory perception. Nervous system dysregulation may represent the common denominator accounting for frequent co-morbidities shared by depression, migraine, and PTSD. Further, it challenges the intellectual paradigm that psychosomatic symptoms are psychological, or “all in the head”, and suggests they are instead the result of a new physiological baseline characterized by dysregulated autonomic circuitry and hormonal responses from chronic stress. A nervous system wired for threat is unable to maintain its important functions for repair, restoration, and health. Given the poor, long-term health consequences associated with chronic stress, nervous system dysregulation in children may act as a harbinger of illness and disease in adulthood, as was identified in the original ACE study [8]. Further research is necessary to test the ANS and HPA-axis in children to determine if routine testing of these systems will illuminate the biological imprint of a potentially common, yet invisible, disorder within the nervous system.

## Figures and Tables

**Table 1:** Group A: Symptom profiles of patients with ≥4 medically unexplained symptoms and total adverse childhood experiences (ACE) scores.

Patient (*n* = 17)	Age (Years)	Sex	Medically-Unexplained Symptoms						Other Medical Problems	Duration of Medical Symptoms	Total ACE Score
			Executive Dysfunction	Sleep Problems	Autonomic Symptoms	Emotional Dysregulation	Digestive/Urinary Problems	Somatization			
1	14	F	Attention problems	Insomnia	None	Anxiety, panic attacks	Constipation, frequent urination	Tremor, weakness	Asthma, chronic fatigue	6 years	3
2	8	F	Poor memory and attention	Nightmares	Dizziness	Cries easily, temper tantrums	None	Headache, blurred vision		4-months	3
3	14	M	Poor attention	Insomnia	Dizziness, syncope	Anxiety, depression, self-harm, suicidality	Diarrhea	Headache weakness, paresthesias	Sinus bradycardia, chronic fatigue	8-months	2
4	15	F	Poor memory and attention	Insomnia, frequent night wakings	Dizziness, syncope,	Anxiety, depression, panic attacks	Constipation	Headache, weakness, paresthesias, abdominal pain	Asthma, cardiac rhythm disturbance, chronic fatigue	4-months	2
5	15	F	Poor concentration	Insomnia	Dizziness	Anxiety, OCD, depression, panic attacks	Reflux, diarrhea	Headache, paresthesias, abdominal pain	Asthma	18-months	1
6	7	M	Poor attention	Insomnia, frequent night wakings	Dizziness	Anxiety, fearfulness	Constipation, nocturnal enuresis	Headaches, blurred vision, paresthesias, weakness	Chronic fatigue	3-years	4
7	13	F	Poor memory and attention	Insomnia, frequent night wakings	Dizziness	Anxiety, depression, panic attacks, self-harm, suicidality	None	Headaches, blurred vision, PNES	Eating disorder, chronic fatigue	4-years	5
8	13	F	Poor attention	Insomnia, nightmares	Migraine	Anxiety, depression, rage and panic attacks	Constipation, vomiting, frequent urination	Abdominal pain	Asthma, tics	8-years	0*
9	5	M	Poor attention	Frequent night wakings, nightmares	None	Anxiety, fear, temper tantrums	Diarrhea, nocturnal enuresis	Headaches, abdominal pain	Asthma, chronic fatigue	2-years	6
10	17	F	Poor memory and attention	Insomnia, night sweats	Dizziness, syncope	Anxiety, depression, anorexia, self-harm	Diarrhea, constipation	Headaches, weakness, PNES	Eating disorder, POTS, sleep apnea, chronic fatigue	3-years	3
11	5	M	Poor memory and attention	Insomnia, frequent night wakings	None	Anxiety, fear, OCD, separation anxiety	None	Headaches	Sleep apnea, tics	3-years	4
12	17	F	Poor memory, slow processing	Insomnia	Dizziness	Anxiety, depression, panic attacks	None	Headaches, blurred vision, unsteady gait, chest pain, tinnitus	Frequent ear infections, chronic fatigue	2-years	2
13	15	F	Poor memory, slow processing	Insomnia	Dizziness	Anxiety, depression, panic attacks	Diarrhea, constipation	Headaches, numbness, pelvic pain	Asthma, chronic fatigue	1-year	0*
14	15	F	Poor memory	Insomnia, frequent night wakings	Dizziness, syncope	Anxiety, depression	Nausea	Headaches, numbness, neck pain, unsteady gait	POTS, chronic fatigue	18-months	2
15	13	M	Poor attention	Insomnia	Dizziness	Panic attacks, rage attacks	Nausea, reflux	Headaches, blurred vision, paresthesias	Derealization, depersonalization,	10-months	4
16	16	F	None	Insomnia, nightmares	Dizziness, migraine	Anxiety, depression, panic and rage attacks, suicidality	Diarrhea, dysuria	Neck pain, back pain	Asthma, eating disorder, heart palpitations, chronic fatigue	5-years	5
17	10	F	Poor attention	Insomnia, nightmares	None	Rage attacks, panic attacks	None	Headaches, abdominal pain, blurred vision	Heart palpitations, shortness of breath, tics	5-years	5

Abbreviations: F = Female, M = Male, PNES—psychogenic non-epileptic events, POTS—Postural orthostatic tachycardia syndrome, OCD—obsessive compulsive disorder. * Other adverse childhood experience, not included the ACE score (homeless, foster care, near-drowning accident).

**Table 2:** Group B: Patients with adverse childhood experiences with 0–3 medically unexplained symptoms.

Patient (*n* = 27)	Age (Years)	Sex	Medically-Unexplained Symptoms						Other Medical Problems	Duration of Medical Symptoms	Total ACE Score
			Executive Dysfunction	Sleep Problems	Autonomic Symptoms	Emotional Dysregulation	Digestive/Urinary Problems	Somatization			
1	16	M	Cognitive dysfunction	None	None	None	None	None	Sickle cell disease, stroke, moyamoya syndrome	2 years	1
2	10	F	None	None	Dizziness, syncope	Depression	None	Headaches, leg pain	None	2-months	1
3	14	F	None	None	None	Depression	None	None	Generalized epilepsy	1-year	1
4	14	F	None	None	Migraine	Depression	None	None	Epilepsy	2-years	1
5	7	M	None	None	None	None	None	Daily headache	None	1 year	0 *
6	11	M	Attention problems	None	None	None	None	None	Tics	5 years	1
7	12	F	None	Insomnia	None	Anxiety, depression, OCD	None	None	Trichotillomania tics, asthma, eczema	7 years	1
8	17	M	Attention problems	None	Migraine	None	None	None	None	5 years	2
9	15	M	Cognitive dysfunction	Insomnia	None	Depression, anxiety	None	None	Epilepsy	3 years	1
10	13	F	None	Insomnia	None	None	None	Headache	None	1 year	0 *
11	9	M	None	Insomnia	None	Depression	None	Headache	Congenital heart disease	3-years	1
12	14	F	None	None	None	None	None	Headache	Breast discharge	1-year	1
13	15	F	None	None	Migraine	None	None	None	None	7-years	1
14	17	M	None	None	None	None	None	None	Epilepsy	1-month	1
15	7	M	Cognitive dysfunction, attention problems	None	None	None	Constipation	None	Developmental delay	7 years	2
16	8	F	None	None	Migraines	Anxiety	None	None	None	3-months	2
17	5	F	None	None	None	None	Constipation	Headache, abdominal pain	None	8-months	1
18	8	F	None	None	Migraine	None	None	None	None	3-months	1
19	5	F	None	None	None	None	None	None	Febrile seizures	2 years	2
20	11	M	Cognitive dysfunction	Insomnia	None	None	None	None	Epilepsy	1-year	2
21	15	F	None	None	No	None	None	None	Epilepsy, Moyamoya disease	3-years	2
22	8	M	None	Insomnia	Migraine	Anxiety	None	None	Cerebral dysgenesis	7-years	1
23	15	M	Cognitive problems, memory problems	Insomnia	None	Anxiety	None	None	Epilepsy	5-months	2
24	13	M	Learning problems, attention problems	Insomnia	Migraine	None	None	None	Neuro-fibromatosis Type-1	6 years	0 *
25	15	F	Cognitive difficulties	None	Dizziness	Anxiety, depression, panic attacks	None	None	None	1-year	1
26	10	F	None	None	None	None	None	Headache	Arnold Chiari malformation	1-year	1
27	10	M	Cognitive dysfunction	Insomnia	None	None	None	Back pain	Cerebral palsy	6-months	1

* Other adverse childhood experience, not included the ACE score (motor vehicle accident, death of a loved one).

**Table 3:** Comparison of adverse childhood experiences between patients with ≥4 medically unexplained symptoms (Group A) and 0–3 medically unexplained symptoms (Group B).

Adverse Childhood Experience	Total (%) *N* = 100	Group A (%) *n* = 17	Group B (%) *n* = 27	OR (95% CI) [Comparing Group A to Group B]	*p*-Value
Physical abuse	2 (2%)	2 (12%)	0	indeterminate	
Emotional/verbal abuse	15 (15%)	10 (59%)	5 (19%)	6.29 (1.60–24.73)	0.009 *
Sexual abuse	5 (5%)	5 (29%)	0	indeterminate	
Physical or emotional neglect	4 (4%)	3 (18%)	1 (4%)	5.57 (0.53–58.69)	0.28
Incarcerated household member	2 (2%)	2 (12%)	0	indeterminate	
Exposure to domestic violence	5 (5%)	4 (24%)	1 (4%)	8 (0.81–79.02)	0.06
Substance user in the home	7 (7%)	7 (41%)	0	indeterminate	
Mental illness in a household member	6 (6%)	4 (24%)	2 (7%)	3.70 (0.59–22.94)	0.19
One or no parents/divorce	37 (37%)	14 (82%)	23 (85%)	0.81 (0.16–4.17)	1.0
Other traumatic events		homeless, foster care, near-drowning accident	motor vehicle accident, death of a loved one		
Median Total ACE score (interquartile range)	0 (0–6)	3 (0–6)	1 (0–2)	N/A	0.0001 ^‡^

Abbreviations: ACE—Adverse Childhood Experience; * Statistically significant using Fisher’s Exact Test; ^‡^ Statistically significant using Two-tailed Mann-Whitney.

## Abbreviations

ACE	Adverse Childhood Experience
ANS	autonomic nervous system
HPA	hypothalamic-pituitary-adrenal
PTSD	post-traumatic stress disorder
